# Genetic overlap of severe psychiatric disorders with lung function and asthma suggests shared biological mechanisms

**DOI:** 10.1192/j.eurpsy.2025.382

**Published:** 2025-08-26

**Authors:** Z. Lu, A. Ploner, P. Jaholkowski, A. Shadrin, B. Brew, O. Andreassen, S. Bergen

**Affiliations:** 1 Karolinska Institutet, Stockholm, Sweden; 2 Oslo University, Oslo, Norway

## Abstract

**Introduction:**

Severe psychiatric disorders, including schizophrenia (SCZ), bipolar disorder (BIP) and anorexia nervosa (AN), are frequently comorbid with lung function decline and asthma. Despite considerable heritability, the genetic relationships between severe psychiatric and respiratory comorbidities are inconclusive.

**Objectives:**

We aimed to thoroughly investigate the shared genetic architecture between three severe psychiatric disorders (SCZ, BIP and AN) and lung function (forced expiratory volume/forced vital capacity ratio) or doctor-diagnosed asthma based on available genome-wide association studies.

**Methods:**

We performed linkage disequilibrium score regression analyses (LDSC) and MiXeR to quantify the genetic overlap. A conditional/conjunctional false discovery rate (cond/conjFDR) approach was employed to detect shared genomic loci. Functional annotations, gene mapping and gene-based enrichment analyses were adopted to explore shared biological mechanisms. Web-based cell-type-specific enrichment analysis (WebCSEA) was employed to identify the cell types enriched for shared genes across tissues. Transcriptome analyses were conducted via S-PrediXcan to prioritize biologically plausible shared genes in relevant tissues.

**Results:**

Despite weak or null genetic correlations identified by LDSC, MiXeR analyses demonstrated extensive moderate polygenic overlap (~400 to 800 shared variants) across all pairs of psychiatric and respiratory phenotypes. The cond/conjFDR approach detected a total of 378 unique loci jointly associated with severe psychiatric disorders and lung function or asthma, harboring a mix of concordant and antagonistic variants. Gene-based enrichment analyses applied to the 4,105 genes mapped to shared loci highlighted cell types including type II pneumocytes and macrophages in lung and monocytes in blood as well as biological processes involving the interferon (IFN) JAK-STAT pathway and natural killer cell activation, suggesting common immune mechanisms. Furthermore, when stratified by respiratory outcome, genes shared with asthma were enriched for immunity-related pathways, whereas genes shared with lung were enriched for non-immune mechanisms, indicating divergent shared etiology. A total of 22 shared genes identified by conjFDR approach were prioritized as biologically plausible in transcriptome analyses.

**Conclusions:**

This study reveals the complicated shared genetic etiology between severe psychiatric disorders and lung function or asthma and implicates overlapping immune and non-immune mechanisms. Our findings suggest that individuals with severe psychiatric disorders should be screened for lung function decline and asthma in clinical settings.

**Disclosure of Interest:**

None Declared

